# CMTM6 expression in M2 macrophages is a potential predictor of PD-1/PD-L1 inhibitor response in colorectal cancer

**DOI:** 10.1007/s00262-021-02931-6

**Published:** 2021-04-05

**Authors:** Xuehui Wu, Xiaoliang Lan, Wanming Hu, Wanning Zhang, Xiangmeng Lai, Shaowan Xu, Jiaoying Li, Weihao Qiu, Wei Wang, Jianbiao Xiao, Feifei Wang, Yanqing Ding, Li Liang

**Affiliations:** 1 Department of Pathology, Nanfang Hospital and Basic Medical College, Southern Medical University, Guangzhou, 510515 Guangdong Province People’s Republic of China; 2 Guangdong Province Key Laboratory of Molecular Tumor Pathology, Guangzhou, 510515 Guangdong Province People’s Republic of China; 3 Department of Pathology, The People’s Hospital of China Three Gorges University, Yichang, 443000 Hubei Province People’s Republic of China; 4 Department of Pathology, Sun Yat-Sen University Cancer Center, Guangzhou, 510060 Guangdong Province People’s Republic of China; 5 Department of Pathology, Zhujiang Hospital, Southern Medical University, Guangzhou, 510280 Guangdong Province People’s Republic of China; 6 General Hospital of Southern Theater Command, People’s Liberation Army of China, Guangzhou, 510010 Guangdong Province People’s Republic of China

**Keywords:** CMTM6, M2 macrophage, PD-L1, Colorectal cancer, Immunotherapy

## Abstract

**Background:**

CMTM6 is a novel key regulator of PD-L1. High expression of both CMTM6 and PD-L1 may predict the benefit of PD-1 axis blockade in lung cancer. We aimed to investigate the expression pattern of CMTM6 between mismatch repair-defective (dMMR) and mismatch repair-proficient (pMMR) colorectal cancer (CRC) tissues and assess its correlation with the response to PD-1/PD-L1 pathway blockade.

**Methods:**

Immunohistochemistry (IHC) was used to analyze CMTM6 and PD-L1 expression and immune cell density in dMMR/pMMR CRC. Quantitative multiplex immunofluorescence (IF) was performed to detect CMTM6, PD-L1, CD4, CD8, CD68 and CD163 expression in CRC patients treated with PD-1/PD-L1 inhibitors.

**Result:**

IHC analysis showed that CMTM6 and PD-L1 were both expressed in tumor cells (TCs) and invasion front immune cells (ICs). CMTM6 and PD-L1 expression and CD4^+^, CD8^+^, CD68^+^ or CD163^+^ cell density were significantly higher in dMMR CRC patients than in pMMR CRC patients. CMTM6 expression was positively correlated with PD-L1 expression and CD163^+^ M2 macrophage density in dMMR CRC. IF analysis showed that the coexpression rate of CMTM6/PD-L1 and the expression rate of CMTM6 in CD8^+^ T cells and CD163^+^ M2 macrophages were significantly increased in the group that exhibited clinical benefit. CMTM6 expression in M2 macrophages was identified as the best biomarker for predicting the responsiveness to PD-1/PD-L1 inhibitors.

**Conclusions:**

CMTM6 expression in M2 macrophages may predict the PD-1/PD-L1 inhibitor response rate in CRC patients more accurately than dMMR/microsatellite instability-high (MSI-H) status. It can also identify pMMR CRC patients who could benefit from PD-1/PD-L1 inhibitors.

**Supplementary Information:**

The online version contains supplementary material available at 10.1007/s00262-021-02931-6.

## Background

Colorectal cancer (CRC) is the third most common cause of cancer-related death in the world, and its mortality rate is increasing in China [1, 2]. CMTM6 is widely expressed in many tissues. It has been reported that CMTM6 is highly expressed in non-small-cell lung cancer, glioma, head and neck squamous cell carcinoma and so on. High expression of CMTM6 correlates with poor prognosis of patients [3–6]. CMTM6 is expressed in advanced non-small-cell lung cancer cells and stromal cells, especially CD68-positive macrophages [7]. Several studies show that CMTM6 maintains the expression of PD-L1 and regulates anti-tumor immunity [8, 9]. Targeting CMTM6 suppresses stem cell-like properties and enhances antitumor immunity in head and neck squamous cell carcinoma [6]. Cancer cell-secreted exosomal CMTM6 induces M2-like macrophage polarization via ERK1/2 signaling pathway [10]. All the data indicate that CMTM6 might be an important target for tumor immunotherapy.

Targeting PD-1/PD-L1 in some tumors has achieved remarkable therapeutic advantages in many early clinical trials, and its prospects are expected to be very promising [11]. Monoclonal antibodies against PD-1/PD-L1 have served in clinical treatment, and many studies show that mismatch repair-defective (dMMR)/microsatellite instability-high (MSI-H) CRC patients have a higher response rate to treatment with PD-1/PD-L1 inhibitors [12, 13]. dMMR/MSI-H has been recognized as a predictive biomarker for the efficacy of anti-PD-1/PD-L1 immunotherapy regardless of tumor type. However, CRC is the one tumor in which immunotherapy has been shown to be less effective, and the majority of CRC patients (particularly mismatch repair-proficient [pMMR] patients) do not benefit from immunotherapy. Although PD-L1 expression, tumor mutational burden (TMB) and tumor-infiltrating lymphocyte density have been reported to predict the efficacy of immune checkpoint inhibitor antibodies in CRC, the predictive value of these biomarkers remains controversial [14, 15]. Thus, it is essential to identify effective biomarkers to help optimize treatment decision-making. Recently, a clinical study showed that high expression of both CMTM6 and PD-L1 is linked to better outcomes in the presence of immune checkpoint inhibitor therapy [7]. We hypothesized that CMTM6 is a potential predictive biomarker for PD-1/PD-L1 inhibitor therapy in CRC. Therefore, we detected the expression pattern of CMTM6 in dMMR and pMMR CRC tissues and determined its predictive value for immunotherapy.

## Materials and methods

### Case select and immunotherapy response assessment

This study was approved by the Institutional Review Boards. Data for a total of 1,328 cases of CRC were gathered between Jan 2015 and Dec 2017 from Nanfang Hospital, Southern Medical University. The inclusion criteria were as follows: (1) all patients were diagnosed with CRC for the first time, and postoperative pathology showed differentiated adenocarcinoma or mucinous adenocarcinoma and signet ring cell carcinoma (Stage I–IV); (2) all patients underwent radical resection of CRC with no less than 10 lymph nodes dissected; (3) no patients received neoadjuvant therapy before operation; (4) no patients received anti-PD-1/PD-L1 therapy. Pathological diagnosis and staging of CRC were performed by the WHO Classification of Tumors of the Digestive System 4th Edition and American Joint Committee on Cancer (AJCC) Staging System. The four mismatch repair proteins (MLH1, MSH2, MSH6 and PMS2) in CRC tissues were identified by immunohistochemistry (IHC) to determine the MMR status. The deletion of MMR proteins was significantly correlated with the patients’ age, tumor size, location, stage and histological classification (*P* < 0.05), but not with gender (*P* = 0.448, Supplementary Table1). After that, 127 pMMR CRC of different genders were randomly selected to match 121 dMMR CRC, in order to make the number of samples in dMMR group and pMMR group generally consistent, and clinicopathological analyses were made in dMMR group (*n* = 121) and pMMR group (*n* = 127).

**Table 1:** Correlation between the expression level of CMTM6 with PD-L1, and CD4^+^, CD8^+^, CD68^+^ or CD163^+^ cells density in dMMR CRC and pMMR CRC

	dMMR (*N* = 121)						pMMR (*N* = 127)					
	CMTM6 TC-	CMTM6 TC +	P/R	CMTM6 IC-	CMTM6 IC +	P/R	CMTM6 TC-	CMTM6 TC +	P/R	CMTM6 IC-	CMTM6 IC +	P/R
*PD-L1 TC*												
−	24	9	*P* < 0.001	24	9	*P* < 0.001	64	24	*P* = 0.488	50	38	*P* = 0.756
+	16	72	*R* = 0.516	3	85	*R* = 0.741	26	13	*R* = 0.062	21	18	*R* = 0.028
*PD-L1 IC*												
−	21	6	*P* < 0.001	21	6	*P* < 0.001	43	9	*P* = 0.015	36	16	*P* = 0.012
+	19	75	*R* = 0.509	6	88	*R* = 0.714	47	28	*R* = 0.217	35	40	*R* = 0.223
*CD4*												
L	16	29	*P* = 0.653	14	31	*P* = 0.074	61	22	*P* = 0.317	50	33	*P* = 0.177
H	24	52	*R* = 0.041	13	63	*R* = 0.163	29	15	*R* = 0.079	21	23	*R* = 0.120
*CD8*												
L	22	41	*P* = 0.650	18	45	*P* = 0.085	75	32	*P* = 0.658	63	44	*P* = 0.119
H	18	40	*R* = 0.041	9	49	*R* = 0.157	15	5	*R* = 0.039	8	12	*R* = 0.139
*CD68*												
L	10	8	*P* = 0.028	8	10	*P* = 0.015	28	12	*P* = 0.884	19	21	*P* = 0.196
H	30	73	*R* = 0.200	19	84	*R* = 0.222	62	25	*R* = 0.013	52	35	*R* = –0.115
*CD163*												
L	5	2	*P* = 0.026	5	2	*P* = 0.001	56	20	*P* = 0.394	46	30	*P* = 0.200
H	35	79	*R* = 0.095	22	92	*R* = 0.292	34	17	*R* = 0.076	25	26	*R* = 0.114

*TC* Tumor cell, *IC* Immune cell, *L* Low density, *H* High density. 121 cases of dMMR and 127 cases pMMR were used for statistical analysis

Samples from a total of 32 patients with metastatic/refractory CRC who were treated with PD-1/PD-L1 inhibitor immunotherapy were harvested at Nanfang Hospital, Zhujiang Hospital, Southern Medical University, General Hospital of Southern Theater Command and the People’s Hospital of China Three Gorges University, and all the samples were taken from the most recent biopsy before immunotherapy. The patients were treated with PD-1/PD-L1 inhibitors (8 patients received camrelizumab, 9 received sintilimab, 12 received toripalimab, 1 received pembrolizumab and 2 received nivolumab) and were followed up for more than 12 weeks, as recommended by the Response Evaluation Criteria in Solid Tumors (RECIST1.1) [16]. The criteria defined clinical benefit as a complete response (CR), a partial response (PR) or stable disease (SD) lasting 12 weeks and defined no clinical benefit as progressive disease (PD) [16].

### IHC

All paraffin blocks were made into unstained slides (3-μm thick) for IHC. The primary antibodies included those against MLH1 (monoclonal mouse, ES05, Dako, Denmark), MSH2 (monoclonal mouse, FE11, Dako, Denmark), MSH6 (monoclonal rabbit, EP49, Dako, Denmark), PMS2 (monoclonal rabbit, EP51, Dako, Denmark), BRAF V600E(monoclonal mouse, VE1, Roche, Switzerland), CMTM6 (monoclonal mouse, RCT6, Absea, China), PD-L1 (monoclonal rabbit, E1 L3N, Cell Signaling Technology, USA), CD4 (monoclonal rabbit, EP204, zsbio, China), CD8 (monoclonal mouse, 1G2B10, Proteintech, USA), CD68 (monoclonal mouse, 3A9A7, Proteintech, USA) and CD163 (monoclonal rabbit, EPR19518, Abcam, USA). All antigens were retrieved with citric acid (pH 6.0) or EDTA (pH 9.0) by heating in a pressure cooker and detected by PV-6000 staining. All procedures were conducted in accordance with the IHC protocol for each antibody.

Cells with expression of all four MMR proteins were considered to be pMMR, and cells with deletion of one or more of these proteins were considered to be dMMR [17]. The immunohistochemical staining of CMTM6 was scored as follows: The extent of staining was scored as 0 (≤ 5%), 1 (6–25%), 2 (26–50%), 3 (51–75%) or 4 (> 75%), and the intensity of staining was scored as 0 (negative), 1 (weak), 2 (moderate) or 3 (strong) [5, 18]. The expression score was obtained by multiplying the intensity and extent scores. Based on previous studies, tumor samples were defined as PD-L1 positive when > 5% of the tumor cells (TCs) and/or tumor-infiltrating ICs were positive for PD-L1 with moderate or strong intensity [19]. The average CD4^+^, CD8^+^, CD68^+^ or CD163^+^ cell density (cells/HPF) was computed across up to five high-power fields (HPFs) in the area with a high cell density [20]. A receiver operating characteristic (ROC) curve was used to evaluate the optimal cutoff value for ICs (CD4^+^, CD8^+^, CD68^+^ and CD163^+^) in the tumor microenvironment of CRC.

### Quantitative multiplex immunofluorescence (IF) staining

Quantitative multiplex IF staining of CMTM6, PD-L1, CD4, CD8, CD68 and CD163 was performed with Opal™ 4-Color Manual IHC kits in the PD-1/PD-L1 inhibitor therapy group (*n* = 32). EDTA (pH 9.0) was utilized to retrieve the antigen in a pressure cooker for 20 min. Tissue sections were treated with blocking buffer and incubated in a humidified chamber for 30 min at room temperature. Then, the blocking buffer was drained off, and primary antibody working solution (4 °C, overnight) was applied. Isotype-specific horseradish peroxidase (HRP)-conjugated secondary antibodies and tyramide-based amplification systems were used only for signal detection. Finally, DAPI working solution was applied for 5 min at room temperature in a humidity chamber. Positive control and negative control slides were included in each round of quantitative multiplex IF staining.

### Bioinformatics analysis of CMTM6 with immune functions in CRC

RNA expression data for 568 CRC samples were acquired from The Cancer Genome Atlas (TCGA) (https://portal.gdc.cancer.gov/). Gene set variation analysis (GSVA) was used to predict the pathway change of different CMTM6 expression. "CIBERSORT" was used to calculate tumor-infiltrating immune cells (TIICs) in high expression group and low expression group. The Pearson correlation was used to investigate the correlation between CMTM6 mRNA expression and some immune genes mRNA expression in CRC.

### Statistical analysis

SPSS 22.0 was used for data analysis. The correlations of CMTM6 and PD-L1 expression with CD4^+^, CD8^+^, CD68^+^ or CD163^+^ cell density were assessed with the χ2 test or the Spearman rank test as described. The cutoff value for defining high-density and low-density CD4^+^, CD8^+^, CD68^+^ and CD163^+^ cells in CRC was assessed by ROC curve analysis. The Kaplan–Meier method was used to estimate the survival distribution, and the differences in progression-free survival (PFS) were analyzed with the log-rank statistic. Differences with *P* < 0.05 were considered statistically significant.

## Results

### CMTM6 and PD-L1 expression and immune cell density in dMMR and pMMR CRC tissues

Four MMR proteins (MLH1, MSH2, MSH6 and PMS2) were assessed by IHC in 1,328 CRC tissues to determine the MSI status. The results showed that 121 cases were cited as dMMR CRC (9.11%). The four most common types of MMR protein changes were codeletion of PMS2 and MLH1 (51.2%, 62/121), codeletion of MSH2 and MSH6 (18.2%, 22/121), deletion of MSH2 (10.7%, 13/121) and deletion of PMS2 (7.4%, 9/121, Supplementary Fig. 1). Correlation analysis showed that MMR status was related to age, tumor size, tumor location, tumor stage and histological classification (*P* = 0.002, *P* < 0.001, *P* < 0.001, *P* < 0.001 and *P* < 0.001, respectively, Supplementary Table 1). The univariate analysis revealed that the PFS of pMMR CRC patients (mean 32.8 months, 95%CI 30.5–35.1 months) was significantly worse than that of dMMR patients (mean 41.7 months, 95%CI 39.5–44.0 months, *P* = 0.006, Supplementary Fig. 2). In our study, 72 cases had loss of MLH1 expression and BRAF V600E mutation was detected by immunohistochemistry. We found that 18 cases (25%) had loss of MLH1 expression in the presence of BRAF V600E mutation, suggesting sporadic dMMR rather than germline mutations. We conducted a clinicopathological analysis of patients and found that BRAF V600E mutation was not related to clinical parameters, expression of CMTM6 and PDL1 and density of CD4^+^, CD8^+^, CD68^+^ and CD163^+^ cells (Supplementary Table 2).

**Fig. 1:**
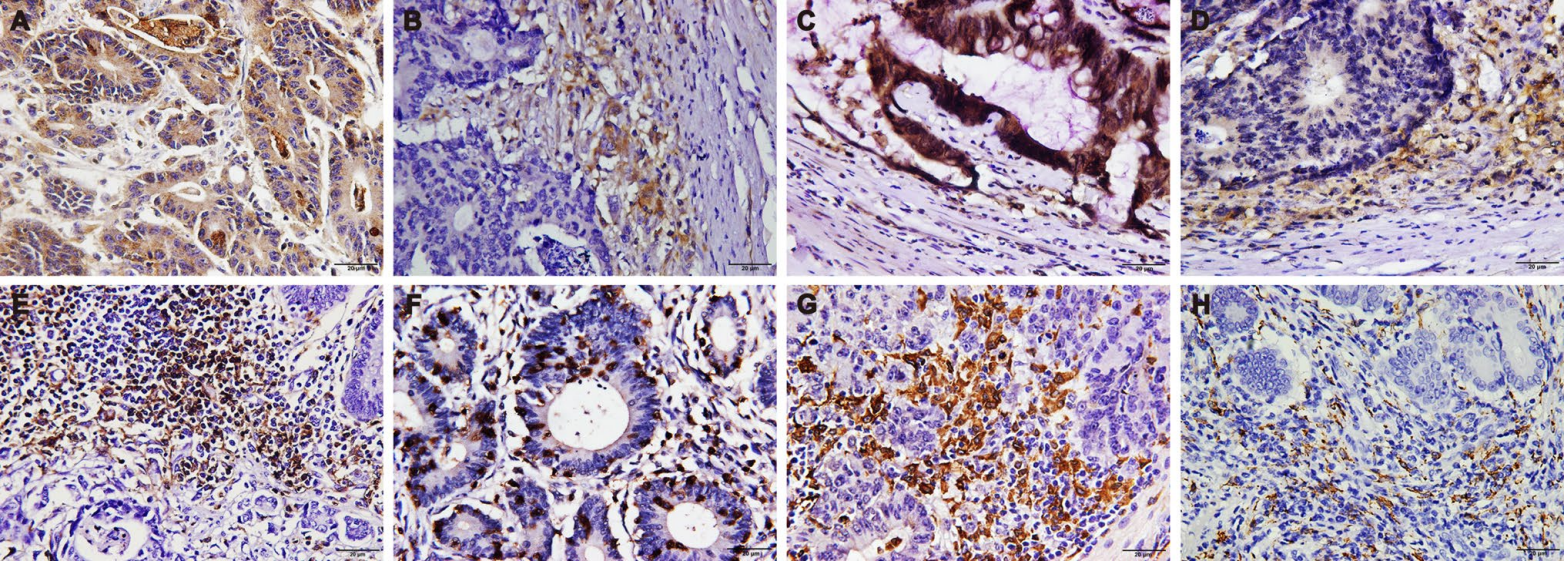
Expression patterns of CMTM6, PD-L1, CD4, CD8, CD68 and CD163 in CRC. **a**, **b** Expression pattern of CMTM6 in tumor cells and/or interstitial immune cells. **c**, **d** Expression pattern of PD-L1 in tumor cells and/or interstitial immune cells. **e** CD4^+^ Cells in tumor microenvironment; **f** CD8^+^ cells in the tumor gland and tumor microenvironment; **g** CD68^+^ cells in tumor microenvironment; **h** CD163^+^ cells in tumor microenvironment. Objective × 40

**Fig. 2:**
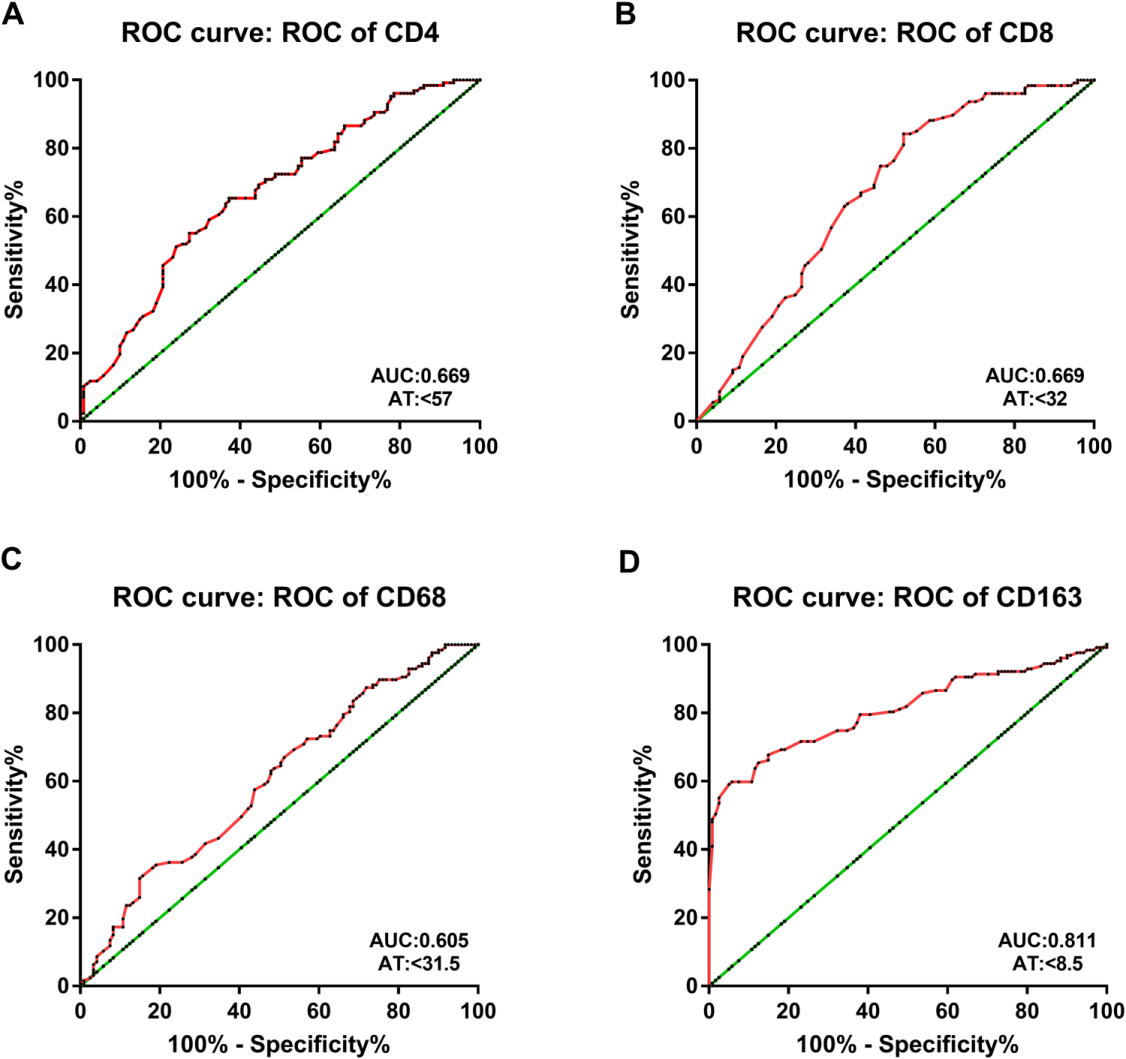
ROC curve of the average density of CD4^+^, CD8^+^, CD68^+^ and CD163^+^ cells; AT represents the value of the best cutoff point; AUC represents the maximum area under the curve

Then, we assessed the expression patterns of the CMTM6, PD-L1, CD4, CD8, CD68 and CD163 proteins in 248 cases of CRC [dMMR (n = 121) and pMMR (n = 127)]. Similar to PD-L1, CMTM6 was expressed in both CRC tumor cells (TCs) and interstitial ICs (Fig. 1a–d). In the dMMR group, the expression rates of CMTM6 and PD-L1 in TCs were 66.94% (81/121) and 72.73% (88/121), respectively, while those in ICs were both 77.69% (94/121). In the pMMR group, the expression rates of CMTM6 and PD-L1 in TCs were 29.13% (37/127) and 30.71% (39/127), respectively, while those in ICs were 44.09% (56/127) and 59.06% (75/127), respectively (Supplementary Table 3). The expression of both CMTM6 and PD-L1 in dMMR CRC was higher than that in pMMR CRC (*P* < 0.001, *P* < 0.001, *P* < 0.001 and *P* = 0.002, Supplementary Table 4). In addition, the positive signals of CD4^+^ T cells, macrophages (CD68^+^) and M2 macrophages (CD163^+^) were located in the microenvironment of the tumor invasion front, while CD8^+^ T cells were distributed not only in the microenvironment of the tumor invasion front but also within the tumor glands (Fig. 1e–h, Supplementary Table 5). The ROC curve showed that the optimal cutoff points for CD4^+^, CD8^+^, CD68^+^ and CD163^+^ cell density were 57, 32, 31.5 and 8.5 cells/HPF, respectively (Fig. 2). Samples with CD4^+^, CD8^+^, CD68^+^ or CD163^+^ cell densities less than 57, 32, 32 or 9 cells/HPF were defined as the low-density group; otherwise, they were defined as the high-density group. The results showed that high densities of CD4^+^, CD8^+^, CD68^+^ and CD163^+^ cells were mainly observed in dMMR CRC (*P* < 0.001, *P* < 0.001, *P* = 0.001, *P* < 0.001, Supplementary Table 4). The above data suggest that CMTM6 and PD-L1 are highly expressed in dMMR CRC tissues, which predominantly have a high density of lymphocytes and macrophages.

### Clinicopathological analysis of CMTM6 and PD-L1 expression and IC density in CRC

**Fig. 3:**
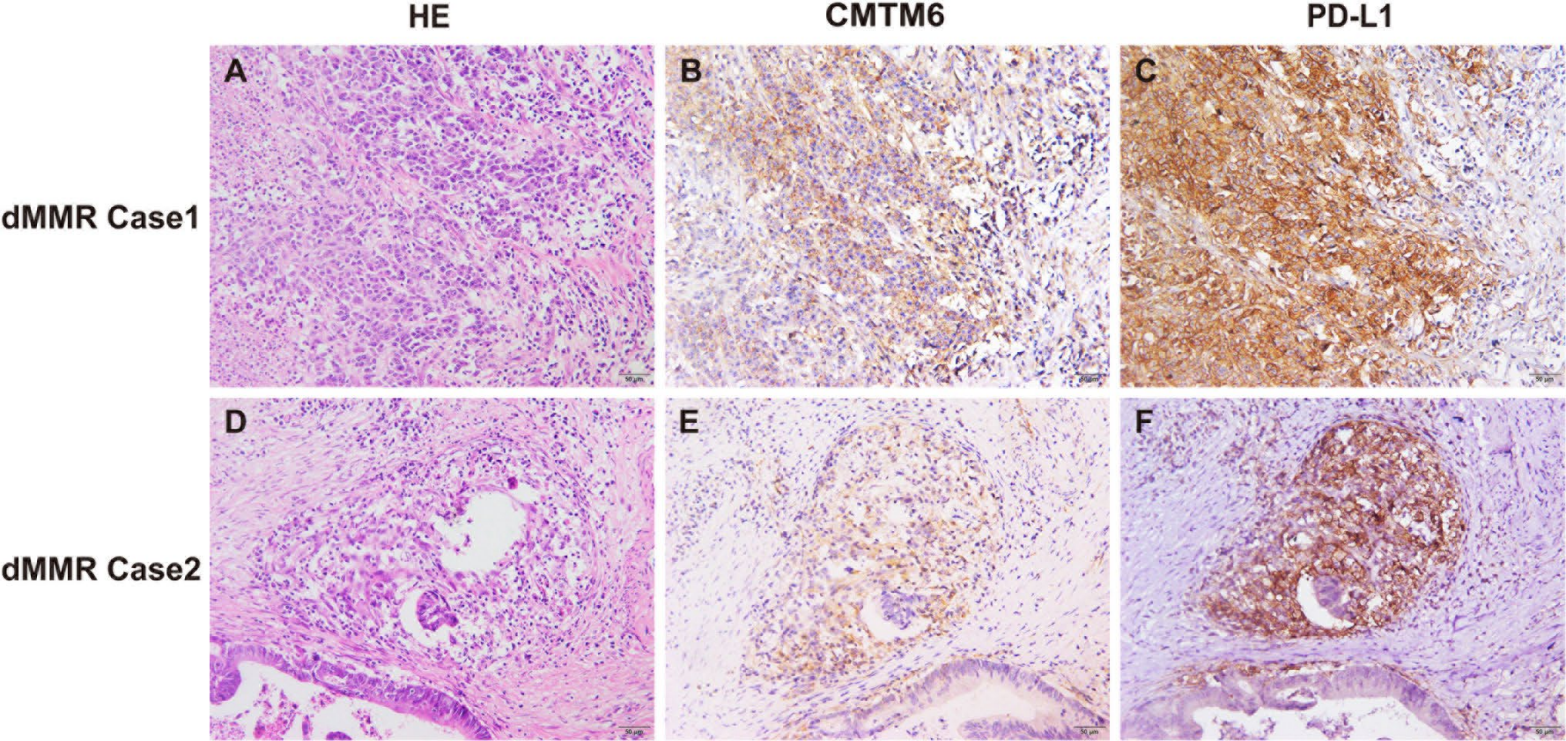
Coexpression of CMTM6 and PD-L1 in dMMR CRC. **a**–**c** dMMR Case1 showed that both CMTM6 and PD-L1 were coexpressed in tumor cells. **d**–**f** dMMR Case2 showed that both CMTM6 and PD-L1 were coexpressed in infiltrating immune cells around the tumor. Objective × 20

We next assessed the correlations between the expression levels of CMTM6 and PD-L1 and the densities of CD4^+^, CD8^+^, CD68^+^ or CD163^+^ cells in dMMR CRC and pMMR CRC. The results showed that the expression levels of CMTM6 and PD-L1 were positively correlated in TCs and ICs (*r* = 0.516, *P* < 0.001 and *r* = 0.714, *P* < 0.001, respectively) in dMMR CRC compared with pMMR CRC (*r* = 0.062, *P* = 0.488 and *r* = 0.223, *P* = 0.012, respectively, Table 1). Coexpression of CMTM6 and PD-L1 in CRC cells and ICs was often observed in dMMR CRC tissues (Fig. 3) but was seldom observed in pMMR tissues (Supplementary Fig. 3). CMTM6 expression in TCs and ICs was positively correlated with CD68^+^ macrophage density (*r* = 0.200, *P* = 0.028 and *r* = 0.222, *P* = 0.015, respectively) and CD163^+^ M2 macrophage density (*r* = 0.095, *P* = 0.026 and *r* = 0.292, *P* = 0.001, respectively) in dMMR CRC but not in pMMR CRC (Table 1). However, the expression of CMTM6 in TCs and ICs was not linked with CD4^+^ or CD8^+^ tumor-infiltrating lymphocyte density in either dMMR or pMMR CRC (Table 1).

Analysis of the correlations of the expression levels of CMTM6 and PD-L1 and the densities of ICs with clinicopathological parameters showed that the expression level of CMTM6 in TCs was related to tumor size in dMMR CRC (*P* = 0.028), and there was no correlation between the expression level of CMTM6 in TCs or ICs and any other clinical parameters in dMMR CRC and pMMR CRC. The expression level of PD-L1 in TCs or ICs was correlated with tumor size in dMMR CRC (*P* = 0.034 and *P* = 0.019, respectively), and the expression level of PD-L1 in TCs was related to tumor size and histological classification in pMMR CRC (*P* = 0.020 and *P* = 0.025), while the expression level of PD-L1 in ICs was related to the age of patients in pMMR CRC (*P* = 0.045). Only high densities of CD4^+^ (*P* < 0.001 and *P* = 0.001) and CD8^+^ (*P* < 0.001 and *P* = 0.015) tumor-infiltrating lymphocytes were associated with tumor stage in both dMMR and pMMR CRC. However, a high density of CD68^+^ and CD163^+^ macrophages was not associated with clinical parameters in either dMMR or pMMR CRC (Supplementary Tables 6–7).

We also evaluated the prognostic value of CMTM6 and PD-L1 expression and IC density in CRC tissues. The results showed that CMTM6 and PD-L1 expression in TCs or ICs was not related to the prognosis of CRC patients, but CRC patients with high densities of CD4^+^ and CD8^+^ lymphocytes had a better prognosis (*P* < 0.001 and *P* = 0.005). Moreover, a high density of CD4^+^ cells was related to a better prognosis for pMMR CRC (*P* = 0.003) but not for dMMR CRC (*P* = 0.404). The densities of CD68^+^ and CD163^+^ macrophages were not related to prognosis in CRC patients (Supplementary Table 8, Supplementary Fig. 4). The results suggest that the expression levels of CMTM6 and PD-L1 are not good indicators for predicting the prognosis of CRC patients.

### The value of CMTM6 in predicting the response to PD-1/PD-L1 inhibitor therapy in CRC patients

We collected data for 32 patients with refractory/metastatic CRC who received PD-1/PD-L1 inhibitors, including 6 dMMR patients and 26 pMMR patients (Supplementary Table 9). Six patients, including two dMMR patients and four pMMR patients, received clinical benefits from immunotherapy. The immunotherapy response rates were 18.8% (6/32) in CRC, 33.3% (2/6) in dMMR CRC and 15.4% (4/26) in pMMR CRC.

The expression of CMTM6 and PD-L1 in CRC patients treated with immunotherapy was detected by IF, and the positive expression rates of CMTM6 and PD-L1 were 59.4% (19/32) and 53.1% (17/32), respectively; however, the coexpression rate of CMTM6 and PD-L1 was 31.3% (10/32). The expression of CMTM6 in ICs was also detected in CRC patients treated with immunotherapy, and CMTM6 was mainly expressed in CD68^+^ macrophages (46.9%, 15/32) and to a lesser degree in CD4^+^ T lymphocytes (21.9%, 7/32), CD8^+^ T lymphocytes (21.9%, 7/32) and CD163^+^ M2 macrophages (18.8%, 6/32, Table 2).

**Table 2:** Expression and coexpression of CMTM6 and PD-L1 and expression of CMTM6 in immune cells (CD4^+^, CD8^+^, CD68^+^ or CD163^+^) in immunotherapy patients of CRC

NO	CMTM6	PD-L1	CMTM6/PD-L1	CMTM6/CD4^+^	CMTM6/CD8^+^	CMTM6/CD163^+^	CMTM6/CD68^+^
1	–	–	–	–	–	–	–
2	+	+	–	–	–	–	+
3	+	+	+	+	+	+	+
4	+	+	+	+	+	+	+
5	–	–	–	–	–	–	–
6	+	+	+	–	–	–	–
7	+	+	+	–	+	+	+
8	+	+	+	+	–	–	+
9	+	+	+	+	–	–	+
10	+	+	–	+	–	+	+
11	–	–	–	–	–	–	–
12	+	+	+	–	+	+	+
13	–	–	–	–	–	–	–
14	+	+	+	–	+	+	+
15	+	+	+	+	–	–	–
16	–	–	–	–	–	–	–
17	+	+	–	–	–	–	+
18	+	–	–	–	–	–	+
19	+	+	+	–	–	+	+
20	–	–	–	–	–	–	–
21	–	–	–	–	–	–	–
22	–	–	–	–	–	–	–
23	+	–	–	–	+	–	–
24	+	+	–	–	–	–	+
25	+	+	–	+	+	–	–
26	+	–	–	–	–	–	+
27	–	–	–	–	–	–	–
28	–	–	–	–	–	–	–
29	+	+	–	–	–	–	+
30	–	+	–	–	–	–	–
31	–	–	–	–	–	–	–
32	–	–	–	–	–	–	–

Then, we examined whether the expression of CMTM6 or PD-L1, coexpression of CMTM6 and PD-L1, or expression of CMTM6 in ICs (CD4^+^, CD8^+^, CD68^+^ and CD163^+^) could predict the responsiveness to PD-1/PD-L1 inhibitors in CRC patients. The results showed that there were significant differences in the coexpression rate of CMTM6 and PD-L1 in TCs and/or ICs and the expression rate of CMTM6 in CD8^+^ T lymphocytes or in CD163^+^ M2 macrophages between the clinical benefit group and the no clinical benefit group (Fisher’s exact test, *P* = 0.006, *P* = 0.012 and *P* = 0.001, respectively, Table 3). However, no meaningful differences were found in the expression of CMTM6 or PD-L1 or the expression of CMTM6 in CD4^+^ T lymphocytes and CD68^+^ macrophages (*P* = 0.361, *P* = 0.178, *P* = 0.590 and *P* = 0.076, respectively).

**Table 3:** Correlation analysis of CMTM6 and PD-L1 expression/coexpression and CMTM6 expression in immune cell (CD4^+^, CD8^+^, CD68^+^ or CD163^+^) with immunotherapy efficacy

	Immunotherapy group (*n* = 32)		*P*
	Clinical benefit group	Non-clinical benefit group	
*CMTM6*			
–	1	12	0.361
+	5	14	
*PD-L1*			
–	1	14	0.178
+	5	12	
*CMTM6/PD-L1*			
–	1	21	0.006
+	5	5	
*CMTM6/CD4*			
–	4	21	0.590
+	2	5	
*CMTM6/CD8*			
–	2	23	0.012
+	4	3	
*CMTM6/CD68*			
–	1	16	0.076
+	5	10	
*CMTM6/CD163*			
–	1	24	0.001
+	5	2	

*CMTM6/PD-L1* Coexpression of CMTM6 and PD-L1, *CMTM6/CD4 and CMTM6/CD8* CMTM6 expression in CD4^+^ or CD8^+^ T lymphocytes, *CMTM6/CD68 and CMTM6/CD163* CMTM6 expression in CD68^+^ or CD163^+^ macrophages or M2 macrophages

**Fig. 4:**
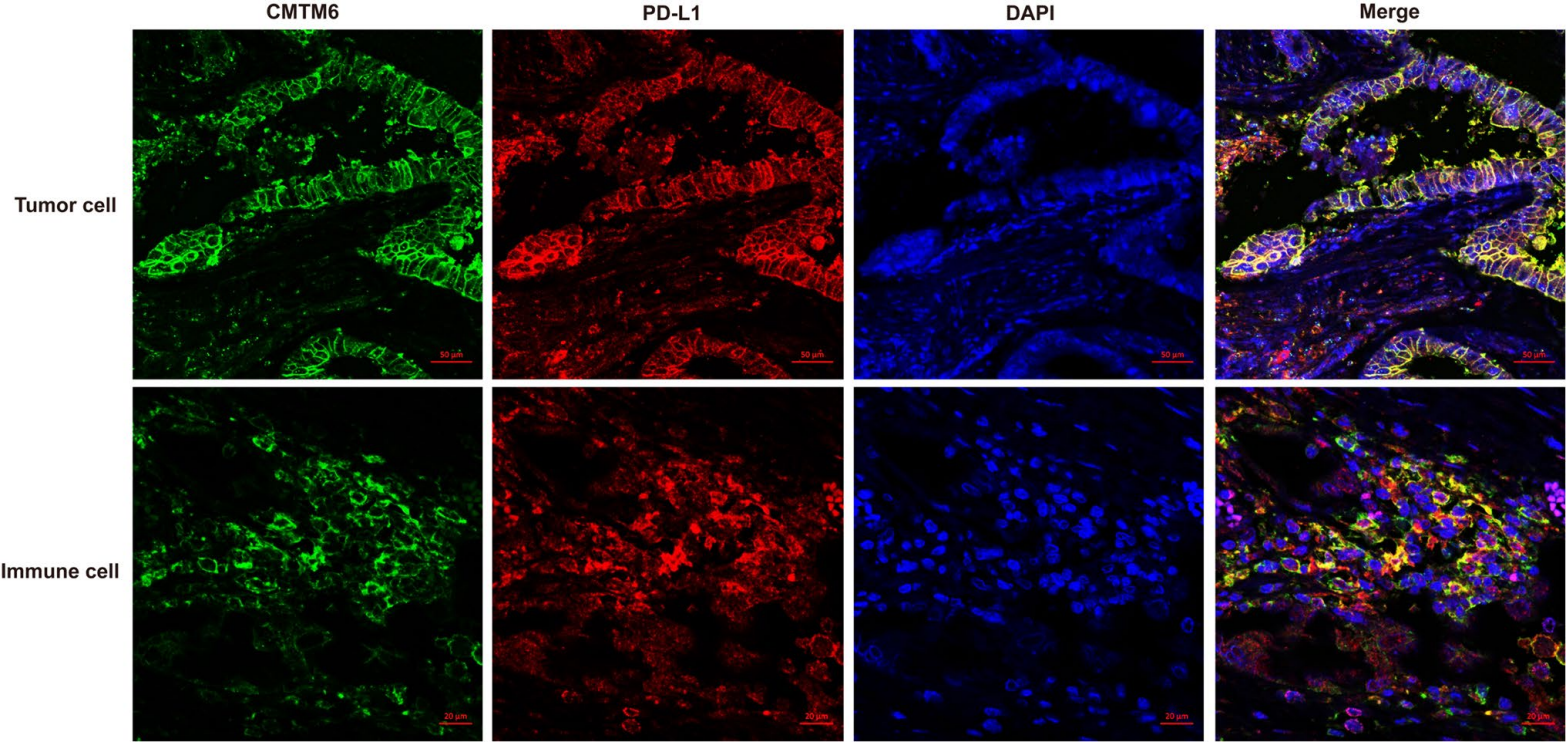
Coexpression of CMTM6 and PD-L1 in the clinical benefit group of CRC. CMTM6 and PD-L1 were mainly coexpressed in tumor cells or interstitial immune cells in the clinical benefit group

**Fig. 5:**
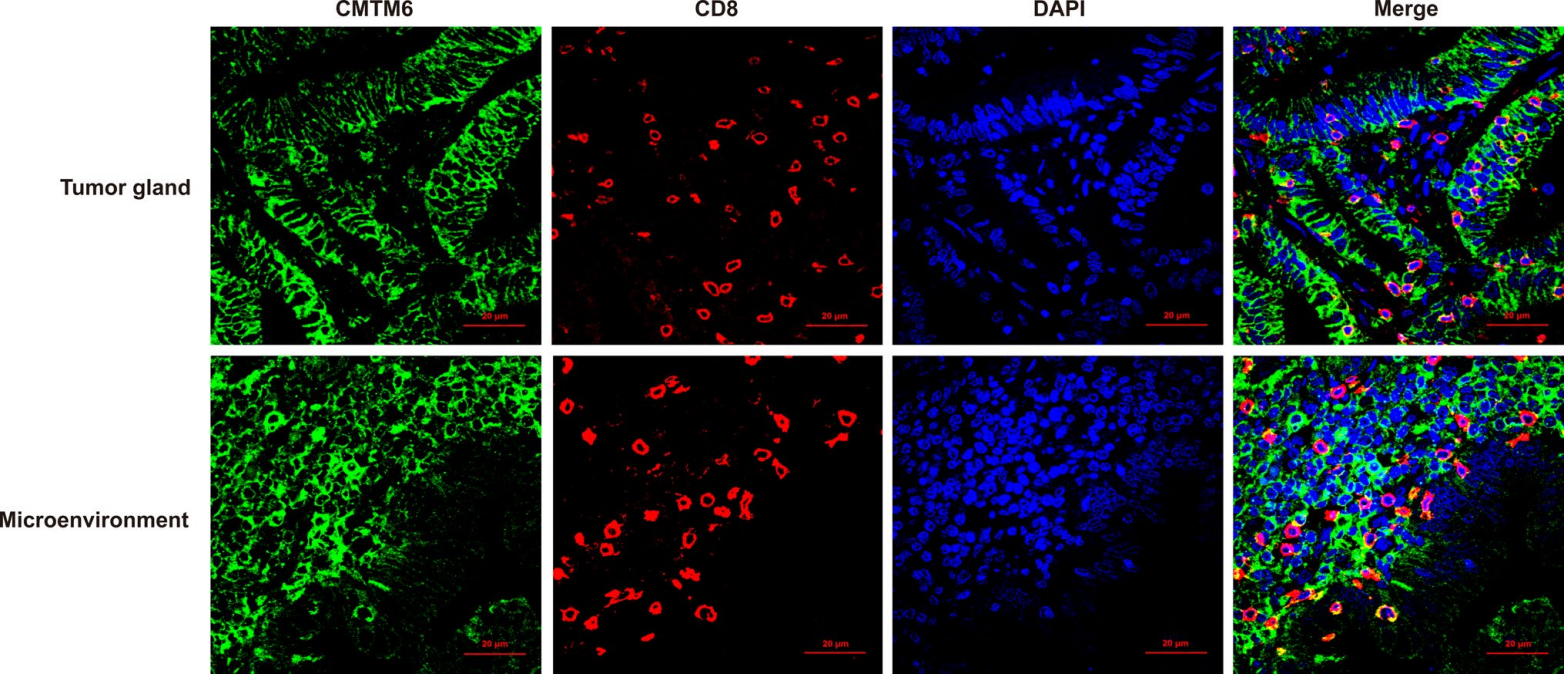
CMTM6 expression in CD8^+^ T lymphocytes in the clinical benefit group of CRC. CMTM6 was mainly expressed in CD8^+^ lymphocytes in tumor gland and tumor microenvironment in the clinical benefit group

**Fig. 6:**
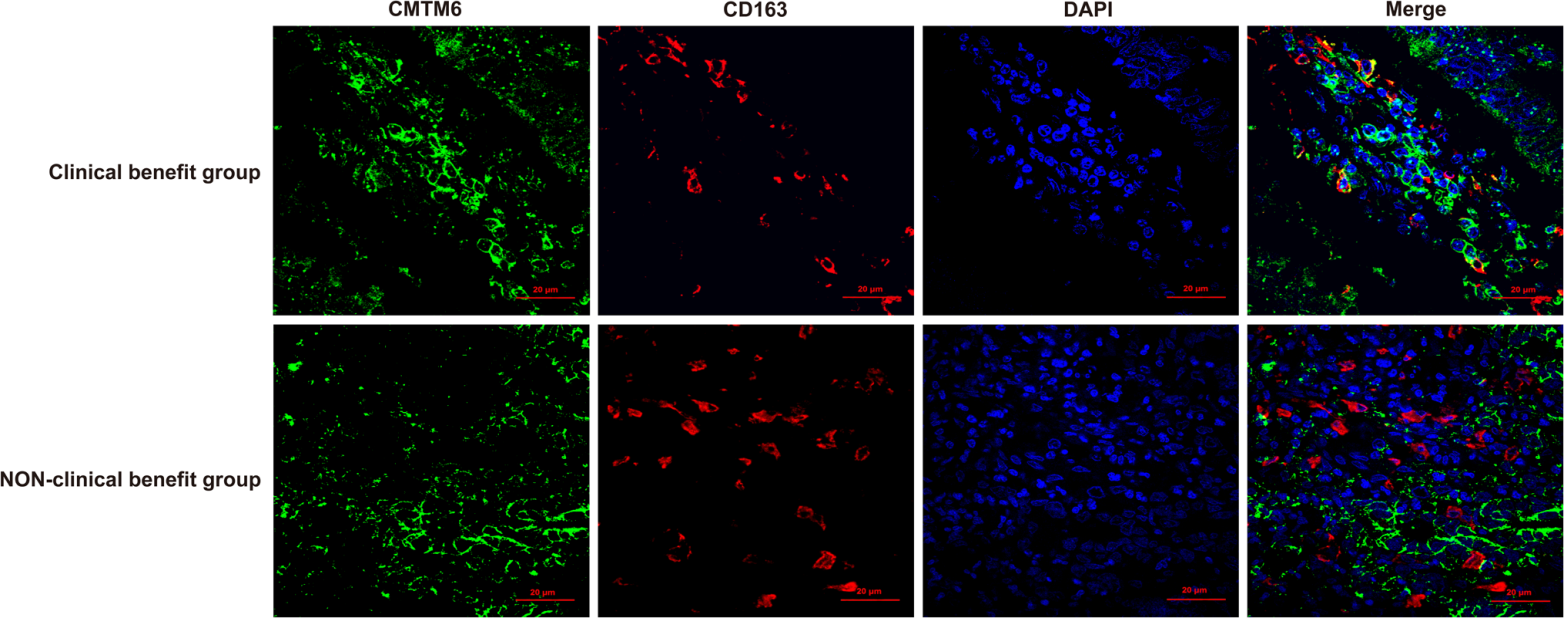
CMTM6 expression in CD163^+^ M2 macrophages in the clinical benefit group and non-clinical benefit group of CRC. CMTM6 was mainly expressed in CD163^+^ M2 macrophages in the clinical benefit group, and rarely in the non-clinical benefit group

Coexpression of CMTM6 and PD-L1 in TCs and/or ICs, expression of CMTM6 in CD8^+^ T lymphocytes and expression of CMTM6 CD163^+^ M2 macrophages was observed in 10, 7 and 7 of 32 patients, respectively, of which 5, 4 and 5 patients experienced clinical benefit, with efficacy rates of 50% (5/10, Fig. 4), 57.1% (4/7, Fig. 5) and 71.4% (5/7, Fig. 6), respectively. Most importantly, the expression of CMTM6 in M2 macrophages predicted the response rate to PD-1/PD-L1 inhibitors in CRC patients (5/7, 71.4%) more accurately than dMMR/MSI-H status (2/6, 33%). The response rate of PD-1/PD-L1 inhibitors predicted according to CMTM6 expression in CD163^+^ M2 macrophages was 66.7% (2/3) in dMMR CRC patients and 75% (3/4) in pMMR CRC patients. Our results indicate that CMTM6 expression in M2 macrophages may be a better predictor of the PD-1/PD-L1 inhibitor response than dMMR/MSI-H status. It can also identify pMMR CRC patients who may benefit from PD-1/PD-L1 inhibitors.

### CMTM6 was closely related to M2 macrophages functions in CRC by bioinformatics analysis

**Fig. 7:**
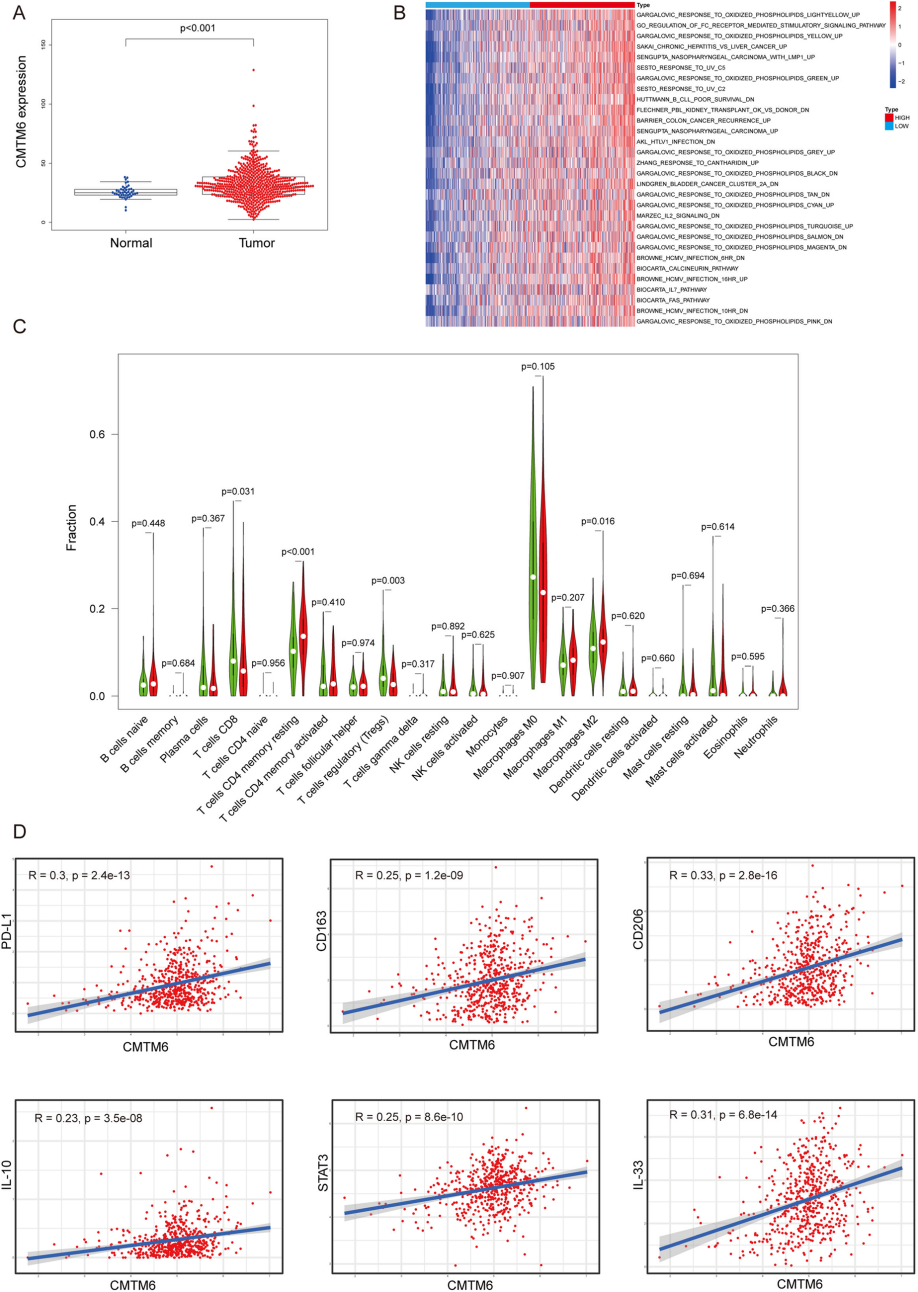
CMTM6 was closely related to immune functions in CRC. **a** CMTM6 was highly expressed in colorectal cancer tissues compared with normal tissue samples (*P* < 0.001). **b** CMTM6 was upregulated in the activating of immune associated pathway and inflammatory response. **c** The relative levels comparison of different immune cell types between CMTM6 low (green) and CMTM6 high (red) group. **d** The correlation analysis between the CMTM6 and M2 macrophage-related genes

We used TCGA public databases to detect the expression of CMTM6 in 568 cases of CRC samples and confirmed that CMTM6 was highly expressed in CRC tissues compared with normal tissue (*P* < 0.001) (Fig. 7a). The gene set variation analysis showed that CMTM6 was up-regulated in the condition of the activation of immune-associated pathway and inflammatory response (Fig. 7b). The CIBERSORT method was then used to evaluate the effect of CMTM6 on the immune cell composition of 568 CRC samples, with the results that high expression of CMTM6 induced the infiltration of CD4 memory resting T cells (*P* < 0.001) and M2 Macrophage (*P* = 0.016), while reduced the proportion of CD8 + T cells (*P* = 0.031) and regulatory T cells (*P* = 0.003) (Fig. 7c). After that, we explored the correlation of CMTM6 expression with some immune genes in CRC by using Pearson Correlation Coefficient. The results further validated that CMTM6 expression was positively correlated with PD-L1 in CRC (*P* < 0.001) (Fig. 7d). Lastly, we used TISIDB website to examine the relationship between CMTM6 expression and M2 macrophage-related gene. CMTM6 expression was positively correlated with CD163 (*P* < 0.001), CD206 (*P* < 0.001), IL-10 (*P* < 0.001), STAT3 (*P* < 0.001), IL-33 (*P* < 0.001) (Fig. 7d). CD163 and CD206 are known markers for M2 macrophage, and cytokines such as IL-10 can regulate the polarization of M2 macrophage by activating STAT3 through IL-10 receptor (IL-10R) [21]. IL-33 is associated with Th2-related cytokines in the IL-1 family that induces M2 macrophage polarization [22]. The above data illustrate that CMTM6 might regulate the polarization and function of M2 macrophage in CRC.

Four hundred and four samples were examined MMR status in the TCGA database. The result showed that CMTM6 was highly expressed in 64 dMMR CRC samples compared with 340 pMMR CRC samples (*P* < 0.001) (Supplementary Fig. 5a) [23]. The CIBERSORT method was used to evaluate the immune cell composition of 41 dMMR CRC samples and 105 pMMR CRC and quantified the immune cell heterogeneity in a mixed cell population (258 samples excluded: CIBERSORT *P* ≥ 0.05). The results showed that there were no types of immune cells affected by CMTM6 expression in dMMR CRC (Supplementary Fig. 5b); however, high expression of CMTM6 induced the infiltration of CD4^+^ memory resting T cells (*P* < 0.001), while reduced the proportion of CD8^+^ T cells (*P* = 0.031) and regulatory T cells (*P* = 0.042) in pMMR CRC (Supplementary Fig. 5c). The above results further revealed the role of CMTM6 in regulating tumor immunology in pMMR CRC.

## Discussion

Anti-PD-1/PD-L1 immunotherapy has achieved great progress in the treatment of certain cancers, such as melanoma [24]**,** non-small cell lung cancer [25]**,** urothelial carcinoma [26]**,** renal cell carcinoma [27] and head and neck squamous cell carcinoma [28]. The US Food and Drug Administration (FDA) approved pembrolizumab as a second- or higher-line treatment for unresectable or metastatic dMMR/MSI-H solid tumors, regardless of the type or location of the tumors [29]. Approximately 50% of dMMR CRC patients show a response to PD-1/PD-L1 immune checkpoint therapy, but the efficacy of immunotherapy is limited in CRC [30, 31]. Only 10–15% of CRC patients have dMMR status [32]**,** and there are no biomarkers to guide immunotherapy in the majority of pMMR CRC patients. Studies have revealed that approximately 10% of pMMR CRC patients respond to PD-1/PD-L1 inhibitors [33, 34]. Therefore, dMMR/MSI-H as a biomarker is not sufficient for predicting the response to immunotherapy in CRC.

High TMB is linked to longer survival in patients with metastatic CRC [35]. TMB is noted in 3% pMMR CRC patients, and whether TMB is beneficial to checkpoint inhibition still needed further study [36]. The FDA approved three IHC assays for PD-L1 to guide treatment decision-making in urothelial carcinoma, melanoma and non-small-cell lung cancer [24, 37, 38]. However, PD-L1 expression in CRC immunotherapy does not appear to be a good predictor of immunotherapy response. Thus, effective biomarkers are still needed to predict the response to immunotherapy in CRC.

The CMTM gene family was first reported by screening databases for sequences and consists of eight members (CMTM1-8) [39]. CMTM6 is a critical protein regulating the stability of PD-L1, and knockdown of CMTM6 reduces the expression of PD-LI, which allows specific T cells to scavenge TCs and enhance the cytotoxic function of T cells [8, 9]. There is a correlation between the expression of CMTM6 and PD-L1 in lung cancer [7, 40]. However, the expression pattern of CMTM6 in CRC was previously unknown. In our study, we found that high expression of CMTM6 was strongly related to PD-L1 expression in dMMR CRC. Moreover, CMTM6 expression in TCs and ICs was positively correlated with CD68^+^ macrophage and CD163^+^ M2 macrophage density in dMMR CRC but not in pMMR CRC. Bioinformatics analysis further showed that CMTM6 expression was positively correlated with PD-L1 in CRC (*P* < 0.001) and CMTM6 expression correlated with M2 macrophage-related gene [CD163 (*P* < 0.001), CD206 (*P* < 0.001), IL-10 (*P* < 0.001), STAT3 (*P* < 0.001), IL-33 (*P* < 0.001)]. In addition, a recent study showed that high expression of both CMTM6 and PD-L1, particularly in stromal ICs (CD68 + macrophages) of non-small-cell lung cancer, might identify the patients with the greatest benefit from PD-1 axis blockade [7]. These results support CMTM6 may predominantly regulate the protein expression of PD-L1 in dMMR CRC tissues, and high expression of CMTM6 may play an important role in the transformation or function of M2 macrophages.

The significance of CMTM6 in tumor prognosis continues to be controversial. Our study showed that the CMTM6 and PD-L1 expression levels were not related to the prognosis of CRC patients. Studies have shown that CMTM6 may serve as an unfavorable prognostic factor in glioma and a favorable prognostic factor in hepatocellular carcinoma [3, 5], and the relationship between the expression of PD-L1 and the prognosis of CRC is still controversial [20, 41]. In addition, our data validated that a high density of CD4^+^ and CD8^+^ lymphocytes was related to a favorable prognosis in CRC (*P* < 0.001 and *P* = 0.005). Moreover, a high density of CD4^+^ cells was related to a favorable prognosis in pMMR CRC but not in dMMR CRC. Some studies have demonstrated that a high density of infiltrating lymphocytes in primary tumors can predict favorable overall survival in CRC patients [42, 43].

Some specific subtypes of patients with immunogenic CRC might benefit from immunotherapy, but biomarkers that can accurately predict the response to treatment are needed. We investigated the value of the expression of CMTM6 or PD-L1, the coexpression of CMTM6 and PD-L1, and their expression in ICs (CD4^+^, CD8^+^, CD68^+^ and CD163^+^) in predicting the responsiveness of CRC patients to PD-1/PD-L1 inhibitors. The results showed that CRC patients with high expression of CMTM6 in CD163^+^ M2 macrophages had the greatest benefit from PD-1/PD-L1 inhibitors, with response rates of 71.4 and 66.7% in dMMR CRC patients and 75% in pMMR CRC patients. However, the PD-1/PD-L1 inhibitor response rate predicted by dMMR status alone was just 33% in our cohort. CMTM6 expression has previously been demonstrated as a promising biomarker that is useful for PD-1/PD-L1 inhibitor therapeutic decision-making in non-small-cell lung cancer [4, 7]. Thus, these data indicate that CMTM6 expression in M2 macrophages may be a reliable predictor for the immunotherapy response in CRC. Bioinformatics analysis is consistent with our experimental results, suggesting that cytokines such as IL-10 can regulate the polarization of M2 macrophages by activating STAT3 through IL-10 receptor (IL-10R). And IL-33 is a cytokine associated with Th2-related cytokines in the IL-1 family that induces M2 polarization.

## Conclusion

Our study shows the exact relationship between the expression of CMTM6 and PD-L1 and M2 macrophage infiltration in CRC tissues. Moreover, CMTM6 expression in M2 macrophages may perform better than MSI status in predicting the response to PD-1/PD-L1 inhibitors in CRC. It can also identify pMMR CRC patients who may benefit from PD-1/PD-L1 inhibitors.

## Supplementary Information

Below is the link to the electronic supplementary material.

Supplementary file1 (PDF 59397 KB)

Supplementary file2 (PDF 860 KB)

Supplementary file3 (PDF 30370 KB)

Supplementary file4 (PDF 4184 KB)

Supplementary file5 (PDF 1431 KB)

Supplementary file6 (PDF 137 KB)

Supplementary file7 (PDF 138 KB)

Supplementary file8 (PDF 62 KB)

Supplementary file9 (PDF 74 KB)

Supplementary file10 (PDF 85 KB)

Supplementary file11 (PDF 152 KB)

Supplementary file12 (PDF 112 KB)

Supplementary file13 (PDF 76 KB)

Supplementary file14 (PDF 140 KB)

## Declarations

### Data availability

All data generated or analyzed during this study are included in this published article and its supplementary information files.
