# Supplementary material for: CMTM6 expression in M2 macrophages is a potential predictor of PD-1/PD-L1 inhibitor response in colorectal cancer
Source: Cancer Immunol Immunother. 2021 Apr 5;70(11):3235–48. doi: 10.1007/s00262-021-02931-6 (PMC8505364; doi:10.1007/s00262-021-02931-6)
Supplement: Supplementary file 1 — Supplementary file1 (PDF 59397 KB) [file 262_2021_2931_MOESM1_ESM.pdf]

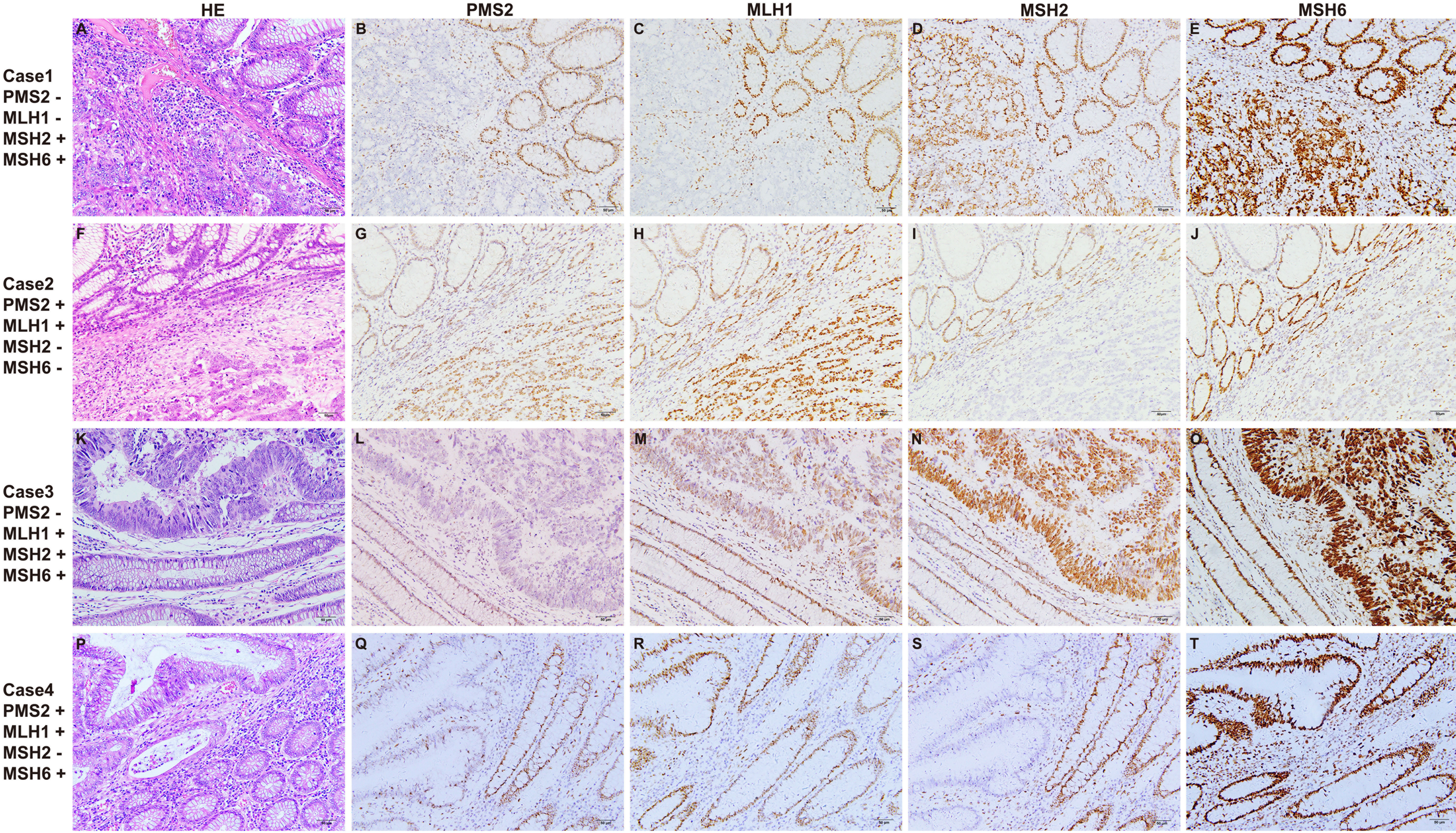

Supplementary Figure1: The most common four types of MMR deletion in the dMMR of colorectal cancer. Case1 showed that both PMS2 and MLH1 were not expressed in colorectal cancer (66/121, 54.5%). **(A)** HE staining, **(B-E)** IHC staining for PMS2 (-), MLH1 (-), MSH2 (+) and MSH6 (+); Case2 showed that MSH2 and MSH6 were not expressed in colorectal cancer (22/121, 18.2%). **(F)** HE staining, **(G-J)** IHC staining for PMS2 (+), MLH1 (+), MSH2 (-) and MSH6 (-); Case3 showed that PMS2 was not expressed in colorectal cancer (12/121, 9.9%). **(K)** HE staining, **(L-O)** IHC staining for PMS2 (-), MLH1 (+), MSH2 (+) and MSH6 (+); Case4 showed that MSH2 was not expressed in colorectal cancer (13/121, 10.7%). **(P)** HE staining, **(Q-T)** IHC staining for PMS2 (+), MLH1 (+), MSH2 (-) and MSH6 (+). Normal intestinal epithelial tissue was used as positive control in each picture. Objective×20.
