# Supplementary material for: CMTM6 expression in M2 macrophages is a potential predictor of PD-1/PD-L1 inhibitor response in colorectal cancer
Source: Cancer Immunol Immunother. 2021 Apr 5;70(11):3235–48. doi: 10.1007/s00262-021-02931-6 (PMC8505364; doi:10.1007/s00262-021-02931-6)
Supplement: Supplementary file 2 — Supplementary file2 (PDF 860 KB) [file 262_2021_2931_MOESM2_ESM.pdf]

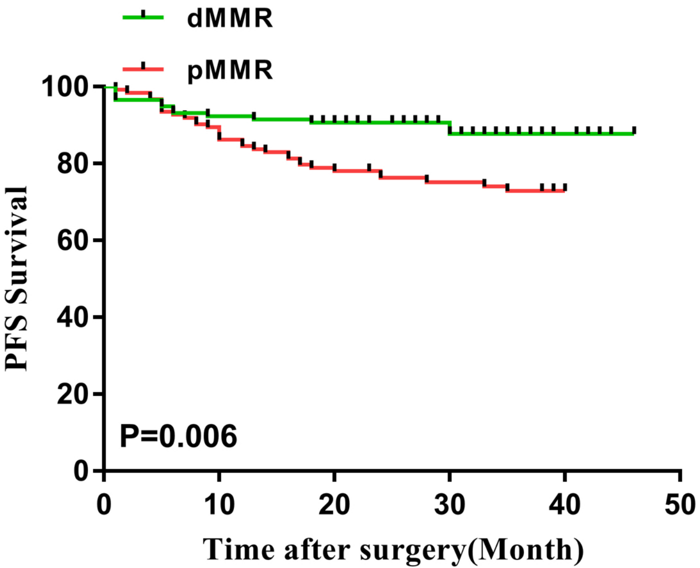


**Supplementary Figure2:** Univariate analysis revealed that the PFS time of dMMR CRC was significantly higher than that of pMMR CRC.
