# Supplementary material for: CMTM6 expression in M2 macrophages is a potential predictor of PD-1/PD-L1 inhibitor response in colorectal cancer
Source: Cancer Immunol Immunother. 2021 Apr 5;70(11):3235–48. doi: 10.1007/s00262-021-02931-6 (PMC8505364; doi:10.1007/s00262-021-02931-6)
Supplement: Supplementary file 3 — Supplementary file3 (PDF 30370 KB) [file 262_2021_2931_MOESM3_ESM.pdf]

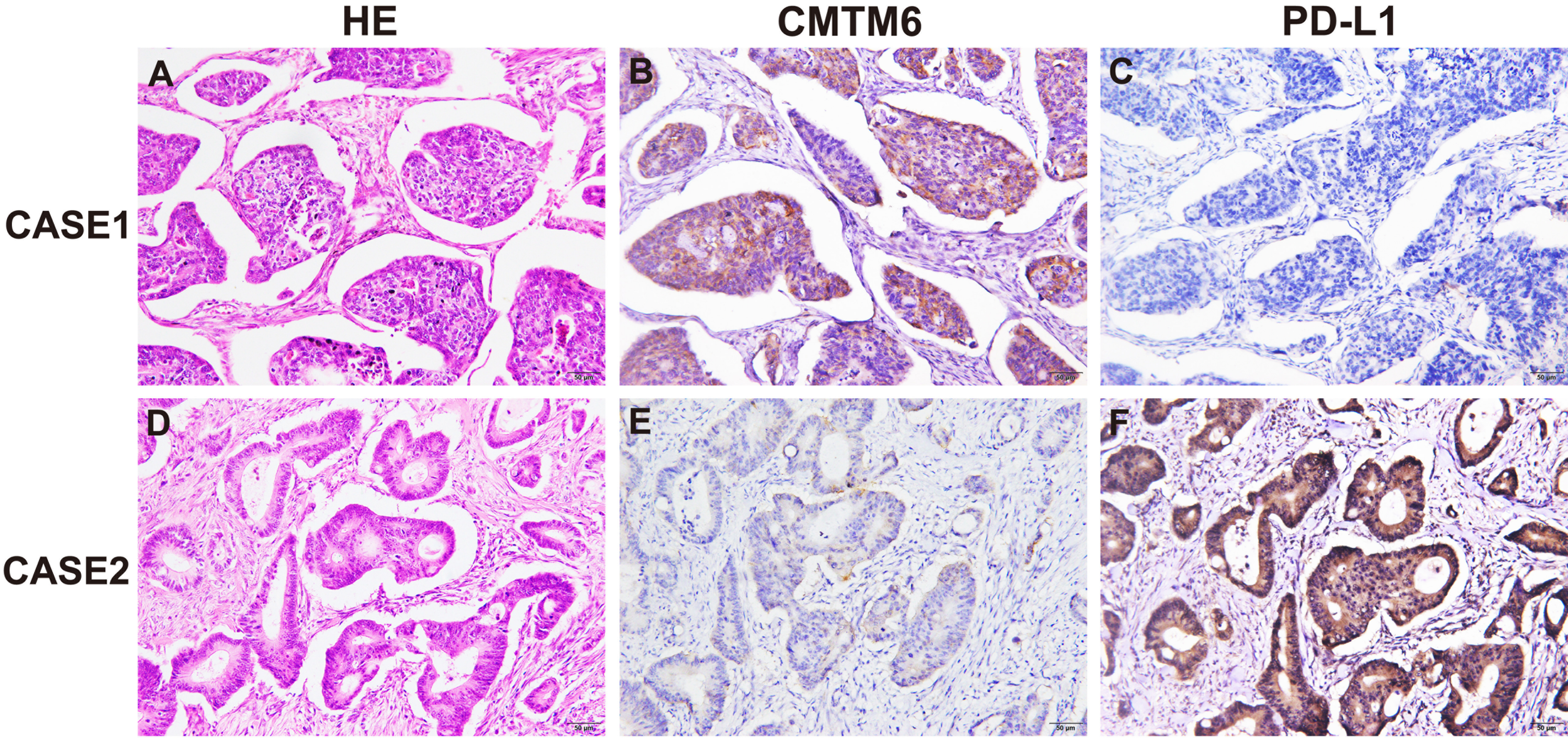

Supplementary Figure3: The expression of CMTM6 and PD-L1 in pMMR CRC. **(A-C)** Case1 showed that CMTM6 was expressed in tumor cells, while PD-L1 was not expressed in tumor. **(D-F)** Case2, CMTM6 was not expressed in tumor, while PD-L1 was highly expressed in tumor cells. Objective × 20
