# Supplementary material for: CMTM6 expression in M2 macrophages is a potential predictor of PD-1/PD-L1 inhibitor response in colorectal cancer
Source: Cancer Immunol Immunother. 2021 Apr 5;70(11):3235–48. doi: 10.1007/s00262-021-02931-6 (PMC8505364; doi:10.1007/s00262-021-02931-6)
Supplement: Supplementary file 4 — Supplementary file4 (PDF 4184 KB) [file 262_2021_2931_MOESM4_ESM.pdf]

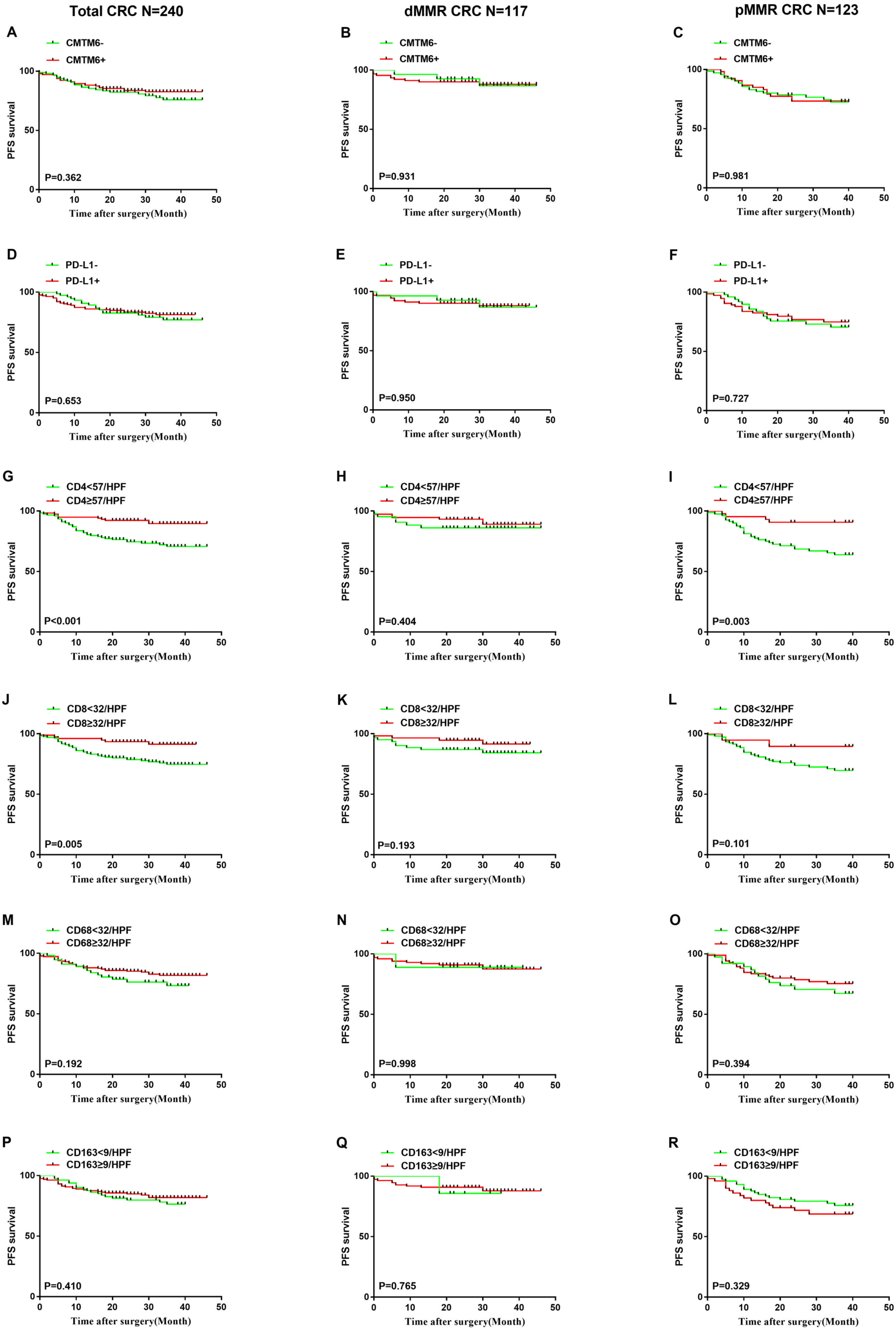

Supplementary Figure4: (A-F) The survival curve showed expression of CMTM6 and PD-L1 in CRC with different MMR subgroups. **(G-R)** The survival curve showed the density of CD4+, CD8+, CD68+ and CD163+ cells in CRC with different MMR subgroups.
