# Supplementary material for: CMTM6 expression in M2 macrophages is a potential predictor of PD-1/PD-L1 inhibitor response in colorectal cancer
Source: Cancer Immunol Immunother. 2021 Apr 5;70(11):3235–48. doi: 10.1007/s00262-021-02931-6 (PMC8505364; doi:10.1007/s00262-021-02931-6)
Supplement: Supplementary file 5 — Supplementary file5 (PDF 1431 KB) [file 262_2021_2931_MOESM5_ESM.pdf]

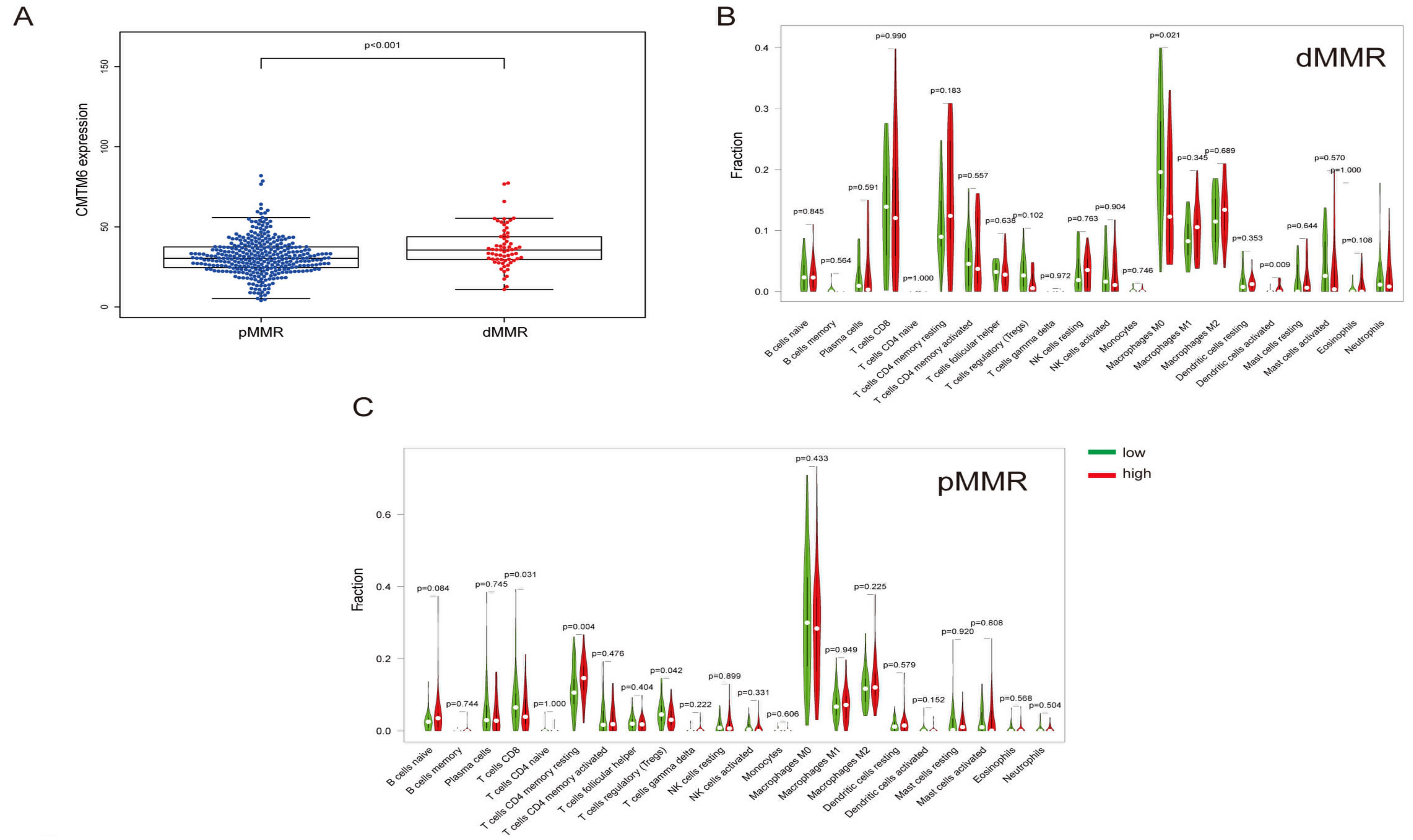

Supplementary Figure5: CMTM6 was closely related to immune functions in pMMR CRC. **(A)** CMTM6 was highly expressed in dMMR CRC compared with pMMR CRC ($p < 0.001$). **(B)** The relative levels comparison of different immune cell types between CMTM6 low(green) and CMTM6 high(red) group of dMMR CRC. **(C)** The relative levels comparison of different immune cell types between CMTM6 low(green) and CMTM6 high(red) group of pMMR CRC.
