# Supplementary material for: CMTM6 expression in M2 macrophages is a potential predictor of PD-1/PD-L1 inhibitor response in colorectal cancer
Source: Cancer Immunol Immunother. 2021 Apr 5;70(11):3235–48. doi: 10.1007/s00262-021-02931-6 (PMC8505364; doi:10.1007/s00262-021-02931-6)
Supplement: Supplementary file 6 — Supplementary file6 (PDF 137 KB) [file 262_2021_2931_MOESM6_ESM.pdf]

Supplementary Table1: Correlation between clinicopathological features with different MMR status of CRC

	Total N=1328	Mismatch repair status		χ2	P value
		dMMR	pMMR		
**Gender**					
Male	854	74	780	0.576	P=0.448
Female	474	47	427		
**Age (year)**					
<50	342	51	291	18.717	P<0.001
≥50	986	70	916		
**Tumor size (CM)**					
<5	560	44	286	9.451	P=0.002
≥5	768	77	921		
**Location**					
Right colon	345	74	271	92.383	P<0.001
Left colon	467	34	433		
Rectum	516	13	503		
**Stage**					
I-II	745	93	652	23.297	P<0.001
III-IV	583	28	555		
**Histological Classification**					
Mucus < 50%	1094	67	1027	66.898	P<0.001
Mucus≥50%	234	54	180		

***121 cases of dMMR and 1207 cases pMMR (all patients)**

	Total N=248	Mismatch repair status		χ2	P value
		dMMR	pMMR		
**Gender**					
Male	158	74	84	0.666	P=0.414
Female	90	47	43		
**Age (year)**					
<50	81	51	30	9.670	P=0.002
≥50	167	70	97		
**Tumor size (CM)**					
<5	119	44	75	12.783	P<0.001
≥5	129	77	52		
**Location**					
Right colon	105	74	31	38.957	P<0.001
Left colon	82	34	48		
Rectum	61	13	48		
**Stage**					
I-II	163	93	70	13.002	P<0.001
III-IV	85	28	57		
**Histological Classification**					
Mucus < 50%	165	67	98	13.217	P<0.001
Mucus≥50%	83	54	29		

***121 cases of dMMR and 127 cases pMMR (random selected)**
