# Supplementary material for: CMTM6 expression in M2 macrophages is a potential predictor of PD-1/PD-L1 inhibitor response in colorectal cancer
Source: Cancer Immunol Immunother. 2021 Apr 5;70(11):3235–48. doi: 10.1007/s00262-021-02931-6 (PMC8505364; doi:10.1007/s00262-021-02931-6)
Supplement: Supplementary file 7 — Supplementary file7 (PDF 138 KB) [file 262_2021_2931_MOESM7_ESM.pdf]

Supplementary table2： Correlation between BRAF mutation detection and clinicopathological features

		BRAF V600E		P
		-	+	
**Gender**	**Male**	33	9	0.408
	**Female**	21	9	
**Age**	**<50**	21	3	0.083
	**≥50**	33	15	
**Tumor size**	**<5cm**	17	6	0.884
	**≥5cm**	37	12	
**Location**	**Right colon**	33	14	0.285
	**Left colon**	16	4	
	**Rectum**	5	0	
**Stage**	**I-II**	43	14	0.867
	**III-IV**	11	4	
**Histological**	**Mucus<50%**	22	10	0.273
**Classification**	**Mucus≥50%**	32	8	
**CMTM6 TC**	**-**	19	4	0.307
	+	35	14	
**CMTM6 IC**	**-**	11	3	0.731
	+	43	15	
**PD-L1 TC**	**-**	11	5	0.513
	+	43	13	
**PD-L1 IC**	**-**	8	3	0.850
	+	46	15	
**CD4**	**L**	17	7	0.564
	**H**	37	11	
**CD8**	**L**	25	9	0.785
	**H**	29	9	
**CD68**	**L**	9	2	0.570
	**H**	45	16	
**CD163**	**L**	3	1	1.000
	**H**	51	17	

TC: tumor cell; IC: immune cell; L: Low density; H: High density
