# Supplementary material for: CMTM6 expression in M2 macrophages is a potential predictor of PD-1/PD-L1 inhibitor response in colorectal cancer
Source: Cancer Immunol Immunother. 2021 Apr 5;70(11):3235–48. doi: 10.1007/s00262-021-02931-6 (PMC8505364; doi:10.1007/s00262-021-02931-6)
Supplement: Supplementary file 8 — Supplementary file8 (PDF 62 KB) [file 262_2021_2931_MOESM8_ESM.pdf]

Supplementary Table3: The expression pattern of CMTM6 and PD-L1 in dMMR CRC and pMMR CRC

	121cases dMMR			127 cases pMMR			
	-	+	+ Percentage (%)	-	+	+ Percentage (%)	p value
**CMTM6 TC**	40	81	66.94%	90	37	29.13%	<0.001
**CMTM6 IC**	27	94	77.69%	71	56	44.09%	<0.001
**PD-L1 TC**	33	88	72.73%	88	39	30.71%	<0.001
**PD-L1 IC**	27	94	77.69%	52	75	59.06%	0.002

TC: tumor cell; IC: immune cell.
