# Supplementary material for: CMTM6 expression in M2 macrophages is a potential predictor of PD-1/PD-L1 inhibitor response in colorectal cancer
Source: Cancer Immunol Immunother. 2021 Apr 5;70(11):3235–48. doi: 10.1007/s00262-021-02931-6 (PMC8505364; doi:10.1007/s00262-021-02931-6)
Supplement: Supplementary file 9 — Supplementary file9 (PDF 74 KB) [file 262_2021_2931_MOESM9_ESM.pdf]

Supplementary Table4: Correlation of CMTM6 and PD-L1 expression and immune ($CD4^+$, $CD8^+$, $CD68^+$ or $CD163^+$) cells density with different MMR status in CRC

	Total	Mismatch repair status		$\chi 2$	P value
		dMMR	pMMR		
**CMTM6 TC**					
**-**	130	40	90	35.513	P<0.001
+	118	81	37		
**CMTM6 IC**					
**-**	98	27	71	29.254	P<0.001
+	150	94	56		
**PD-L1 TC**					
**-**	121	33	88	43.786	P<0.001
+	127	88	39		
**PD-L1 IC**					
**-**	79	27	52	9.908	P=0.002
+	169	94	75		
**CD4**					
**L**	128	45	83	19.681	P<0.001
**H**	120	76	44		
**CD8**					
**L**	170	63	107	29.773	P<0.001
**H**	78	58	20		
**CD68**					
**L**	58	18	40	9.553	P=0.002
**H**	190	103	87		
**CD163**					
**L**	83	7	76	81.318	P<0.001
**H**	165	114	51		

TC: tumor cell; IC: immune cell; L: Low density; H: High density
