# Supplementary material for: CMTM6 expression in M2 macrophages is a potential predictor of PD-1/PD-L1 inhibitor response in colorectal cancer
Source: Cancer Immunol Immunother. 2021 Apr 5;70(11):3235–48. doi: 10.1007/s00262-021-02931-6 (PMC8505364; doi:10.1007/s00262-021-02931-6)
Supplement: Supplementary file 10 — Supplementary file10 (PDF 85 KB) [file 262_2021_2931_MOESM10_ESM.pdf]

Supplementary Table5: Immune cells (CD4^{+}, CD8^{+}, CD68^{+} and CD163^{+}) density (cells/HPF) in dMMR CRC and pMMR CRC

	dMMR(mean,95%CI) N=121	pMMR(mean,95%CI) N=127	Total(mean,95%CI) N=248
**CD4**	111.463(92.41-130.51)	60.969(51.5-70.44)	85.605(74.73-96.48)
**CD8**	35.355(29.91-40.80)	20.803(18.12-23.48)	27.903(24.80-31.01)
**CD68**	66.760(58.20-75.32)	49.504(44.16-54.85)	57.923(52.85-63.00)
**CD163**	38.678(33.37-43.99)	15.598(10.98-20.22)	26.859(23.09-30.63)
