# Supplementary material for: CMTM6 expression in M2 macrophages is a potential predictor of PD-1/PD-L1 inhibitor response in colorectal cancer
Source: Cancer Immunol Immunother. 2021 Apr 5;70(11):3235–48. doi: 10.1007/s00262-021-02931-6 (PMC8505364; doi:10.1007/s00262-021-02931-6)
Supplement: Supplementary file 11 — Supplementary file11 (PDF 152 KB) [file 262_2021_2931_MOESM11_ESM.pdf]

Supplementary Table6: The correlation of the expression of CMTM6 and PD-L1 and immune cells density with clinicopathological parameters in dMMR CRC

dMMR N=121	CMTM6 TC		p	CMTM6 IC		p	PD-L1 TC		p	PD-L1 IC		p	CD4		p	CD8		p	CD68		p	CD163		p
				-	+					-	+		L	H		L	H		L	H		L	H	
**Gender**																								
Male	23	51	0.562	16	58	0.818	21	53	0.732	18	56	0.505	28	46	0.853	37	37	0.568	9	65	0.293	5	69	0.566
Female	17	30		11	36		12	35		9	38		17	30		26	21		9	38		2	45	
**Age(year)**																								
<50	15	36	0.467	10	41	0.542	12	39	0.430	11	40	0.867	14	37	0.058	28	23	0.594	8	43	0.831	4	47	0.408
≥50	25	45		17	53		21	49		16	54		31	39		35	35		10	60		3	67	
**Tumor size**																								
<5cm	20	24	0.028	14	30	0.058	17	27	0.034	15	29	0.019	15	29	0.594	18	26	0.063	8	36	0.440	2	42	0.659
≥5cm	20	57		13	64		16	61		12	65		30	47		45	32		10	67		5	72	
**Location**																								
Right colon	19	54	0.114	12	61	0.113	18	55	0.576	15	58	0.714	23	50	0.219	38	35	0.515	10	63	0.894	3	70	0.326
Left colon	16	19		12	23		10	25		8	27		15	20		20	15		6	29		4	31	
Rectum	5	8		3	10		5	8		4	9		7	6		5	8		2	11		0	13	

**Stage**																								
I-II	30	63	0.733	20	73	0.697	28	65	0.202	23	70	0.244	26	67	<0.001	40	53	<0.001	13	80	0.613	6	87	0.567
III-IV	10	18		7	21		5	23		4	24		19	9		23	5		5	23		1	27	
**Histological Classification**																								
Mucus < 50%	26	41	0.134	17	50	0.368	18	49	0.911	15	52	0.983	26	41	0.682	37	30	0.439	11	56	0.595	5	62	0.379
Mucus≥50%	14	40		10	44		15	39		12	42		19	35		26	28		7	47		2	52	

TC: tumor cell; IC: immune cell; L: Low density; H: High density
