# Supplementary material for: CMTM6 expression in M2 macrophages is a potential predictor of PD-1/PD-L1 inhibitor response in colorectal cancer
Source: Cancer Immunol Immunother. 2021 Apr 5;70(11):3235–48. doi: 10.1007/s00262-021-02931-6 (PMC8505364; doi:10.1007/s00262-021-02931-6)
Supplement: Supplementary file 12 — Supplementary file12 (PDF 112 KB) [file 262_2021_2931_MOESM12_ESM.pdf]

Supplementary Table7: The correlation of the expression of CMTM6 and PD-L1 and immune cells density with clinicopathological parameters in pMMR CRC

pMMR N=127	CMTM6 TC		p	CMTM6 IC		p	PD-L1 TC		p	PD-L1 IC		p	CD4		p	CD8		p	CD68		p	CD163		p
				-	+					-	+		L	H		L	H		L	H		L	H	
**Gender**																								
Male	58	26	0.528	46	38	0.717	56	28	0.370	31	53	0.196	53	31	0.455	72	12	0.527	25	59	0.557	45	39	0.044
Female	32	11		25	18		32	11		21	22		30	13		35	8		15	28		31	12	
**Age(year)**																								
<50	20	10	0.562	19	11	0.348	24	6	0.146	17	13	0.045	24	6	0.054	27	3	0.323	8	22	0.515	23	7	0.031
≥50	70	27		52	45		64	33		35	62		59	38		80	17		32	65		53	44	
**Tumor size**																								
<5cm	51	24	0.393	42	33	0.979	46	29	0.020	27	48	0.174	47	28	0.445	62	13	0.556	24	51	0.883	46	29	0.681
≥5cm	39	13		29	23		42	10		25	27		36	16		45	7		16	36		30	22	
**Location**																								
Right colon	22	9	0.668	15	16	0.625	20	11	0.728	12	19	0.879	20	11	0.559	29	2	0.021	10	21	0.417	14	17	0.146
Left colon	32	16		28	20		35	13		19	29		34	14		43	5		12	36		30	18	
Rectum	36	12		28	20		33	15		21	27		29	19		35	13		18	30		32	16	

**Stage**																								
I-II	48	22	0.528	35	35	0.137	46	24	0.333	25	45	0.184	37	33	0.001	54	16	0.015	21	49	0.688	42	28	0.968
III-IV	42	15		36	21		42	15		27	30		46	11		53	4		19	38		34	23	
**Histological Classification**																								
Mucus < 50%	71	27	0.471	57	41	0.346	63	35	0.025	40	58	0.957	58	40	0.007	80	18	0.136	30	68	0.693	55	43	0.116
Mucus≥50%	19	10		14	15		25	4		12	17		25	4		27	2		10	19		21	8	

TC: tumor cell; IC: immune cell; L: Low density; H: High density
