# Supplementary material for: CMTM6 expression in M2 macrophages is a potential predictor of PD-1/PD-L1 inhibitor response in colorectal cancer
Source: Cancer Immunol Immunother. 2021 Apr 5;70(11):3235–48. doi: 10.1007/s00262-021-02931-6 (PMC8505364; doi:10.1007/s00262-021-02931-6)
Supplement: Supplementary file 13 — Supplementary file13 (PDF 76 KB) [file 262_2021_2931_MOESM13_ESM.pdf]

Supplementary Table8：Prognostic analysis of CMTM6 and PD-L1 expression and immune cells density in CRC

	dMMR N=117			pMMR N=123			Total N=240		
	N	PFS Time (Month) Mean,95%CI	p	N	PFS Time (Month) Mean,95%CI	p	N	PFS Time (Month) Mean,95%CI	p
**CMTM6 TC**									
-	39	43.6(41.0-46.3)	0.370	89	33.1(30.5-35.8)	0.628	127	39.3(36.9-41.7)	0.783
+	78	40.8(37.8-43.9)		34	32.0(27.6-36.3)		113	39.5(36.8-42.1)	
**CMTM6 IC**									
-	27	42.6(38.8-46.3)	0.931	70	32.9(29.8-35.9)	0.981	96	38.6(35.7-41.5)	0.362
+	90	41.5(38.9-44.2)		53	32.7(29.3-36.1)		144	40.0(37.7-42.2)	
**PD-L1 TC**									
-	32	44.4(42.1-46.6)	0.275	86	33.2(30.7-35.8)	0.690	118	39.4(37.0-41.8)	0.751
+	85	39.1(36.3-41.8)		37	31.8(27.2-36.4)		122	37.7(35.2-40.2)	
**PD-L1 IC**									
-	27	42.4(38.4-46.4)	0.950	49	32.7(29.3-36.1)	0.727	75	39.3(36.4-42.3)	0.653
+	90	39.8(37.3-42.3)		74	32.9(29.9-35.9)		165	37.8(35.7-39.9)	
**CD4**									
L	43	40.4(36.2-44.6)	0.404	80	30.5(27.4-33.5)	0.003	123	36.3(33.4-39.1)	<0.001
H	74	42.6(40.1-45.1)		43	37.3(34.6-39.9)		117	42.6(40.7-44.6)	
**CD8**									
L	61	40.2(36.6-43.7)	0.193	104	32.0(29.5-34.6)	0.101	165	37.7(35.3-40.0)	0.005
H	56	40.7(38.4-42.9)		19	36.9(32.7-41.1)		75	40.5(38.4-42.5)	
**CD68**									
L	18	37.1(32.0-42.2)	0.998	38	31.8(27.6-35.9)	0.394	56	33.9(30.5-37.2)	0.192
H	99	41.8(39.4-44.2)		85	33.3(30.6-35.9)		184	39.9(37.9-41.9)	
**CD163**									
L	7	32.6(28.2-37.0)	0.765	73	33.9(31.3-36.6)	0.329	80	34.2(31.7-36.7)	0.410
H	110	41.7(39.4-44.1)		50	31.1(27.2-35.0)		160	39.7(37.5-41.9)	

TC: tumor cell; IC: immune cell; L: Low density; H: High density
