# Supplementary material for: CMTM6 expression in M2 macrophages is a potential predictor of PD-1/PD-L1 inhibitor response in colorectal cancer
Source: Cancer Immunol Immunother. 2021 Apr 5;70(11):3235–48. doi: 10.1007/s00262-021-02931-6 (PMC8505364; doi:10.1007/s00262-021-02931-6)
Supplement: Supplementary file 14 — Supplementary file14 (PDF 140 KB) [file 262_2021_2931_MOESM14_ESM.pdf]

Supplementary Table9: The basic conditions of 32 CRC patients who received PD-1/PD-L1 inhibitor immunotherapy

NO.	Gender	Age (Year)	Diagnosis	MMR Status	PD-1 1inhibitor	efficacy assessment
1	F	50	adenocarcinoma	pMMR	Camrelizumab	PD
2	F	49	adenocarcinoma	dMMR	Camrelizumab	PD
3	M	73	adenocarcinoma	dMMR	Camrelizumab	PR
4	F	72	adenocarcinoma	dMMR	Sintilimab	SD
5	M	60	adenocarcinoma	pMMR	Sintilimab	PD
6	M	51	adenocarcinoma	dMMR	Pembrolizumab	PD
7	F	57	adenocarcinoma	dMMR	Sintilimab	PD
8	F	44	adenocarcinoma	pMMR	Toripalimab	PD
9	F	44	adenocarcinoma	pMMR	Toripalimab	PD
10	M	63	adenocarcinoma	pMMR	Toripalimab	PD
11	M	57	adenocarcinoma	pMMR	Nivolumab	PD
12	F	18	adenocarcinoma	pMMR	Toripalimab	SD
13	M	53	adenocarcinoma	pMMR	Toripalimab	PD
14	F	51	adenocarcinoma	pMMR	Sintilimab	SD
15	M	50	adenocarcinoma	pMMR	Toripalimab	PD
16	M	38	adenocarcinoma	pMMR	Toripalimab	PD
17	F	47	adenocarcinoma	pMMR	Toripalimab	PD
18	F	44	adenocarcinoma	pMMR	Toripalimab	PD
19	F	66	adenocarcinoma	pMMR	Camrelizumab	SD
20	F	49	adenocarcinoma	pMMR	Camrelizumab	PD
21	F	42	adenocarcinoma	pMMR	Nivolumab	PD
22	F	41	adenocarcinoma	pMMR	Camrelizumab	SD
23	M	57	adenocarcinoma	pMMR	Toripalimab	PD
24	M	52	adenocarcinoma	pMMR	Toripalimab	PD
25	M	60	adenocarcinoma	pMMR	Toripalimab	PD
26	M	32	adenocarcinoma	dMMR	Sintilimab	PD
27	M	50	adenocarcinoma	pMMR	Sintilimab	PD
28	F	54	adenocarcinoma	pMMR	Sintilimab	PD
29	M	55	adenocarcinoma	pMMR	Sintilimab	PD
30	F	63	Mucinous adenocarcinoma	pMMR	Camrelizumab	PD
31	M	66	adenocarcinoma	pMMR	Camrelizumab	PD
32	M	67	adenocarcinoma	pMMR	Sintilimab	PD
